# Colchicine, Caffeine, Gramine, and Their Derivatives as Potential Herbicides, Fungicides, and Insecticides

**DOI:** 10.3390/ijms251810081

**Published:** 2024-09-19

**Authors:** Joanna Kurek, Arleta Sierakowska, Natalia Berdzik, Beata Jasiewicz

**Affiliations:** Department of Bioactive Products, Faculty of Chemistry, Adam Mickiewicz University, Uniwersytetu Poznańskiego 8, 61-614 Poznań, Polandnatalia.berdzik@amu.edu.pl (N.B.); beata.jasiewicz@amu.edu.pl (B.J.)

**Keywords:** alkaloids, colchicine, gramine, caffeine, herbicidal activity, insecticidal activity, fungicidal activity

## Abstract

A preliminary in silico screening of 94 compounds, including colchicine, caffeine, gramine, and their derivatives, was conducted to identify potential herbicides, insecticides, and fungicides. Among the compounds tested, only gramine and its 13 derivatives exhibited potential activity. These compounds were further tested against eight species of insects, three species of weeds, and four species of fungi. All of the tested alkaloids were found to be ineffective as herbicides and insecticides, but they did exhibit some fungicidal activity. Four gramine derivatives showed some activity against *Phytophthora infestans*, *Botrytis cinerea*, *Zymoseptoria tritici*, and *Fusarium culmorum*.

## 1. Introduction

Nowadays, the sustainable management of agricultural systems is crucial for both food production and ecological biodiversity [1]. Agroecological research plays a vital role in protecting agricultural systems and crops [2,3], especially against troublesome species of insects, weeds, and fungi. Some common weeds include the annual meadow grass *Poa annua* L., the creeping bent *Agrostis stolonifera* L., and the shepherd’s purse *Capsella* L. *Poa annua* is a common weed in agricultural and urban systems worldwide [4]. It is resistant to commonly used herbicides [4,5,6,7], but efforts have been made to control it [8]. *Agrostis stolonifera* is another highly adaptable weed that can spread rapidly on fertile, moist soils to form extensive mats [9]. Controlling weeds like *Agrostis stolonifera* is critical. In addition to weeds, control of unwanted insects and fungi is essential for agriculture. *Anthonomus* L. insects, such as the boll weevil, strawberry blossom weevil, and pepper weevil, are significant agricultural pests. They infest over 200 tropical fruits and vegetables, causing severe destruction and damage that reduces crop yields and requires costly sorting of fresh produce [10]. The Mediterranean fruit fly (*Ceratitis capitata* L.) is considered one of the most destructive fruit pests globally and the most economically significant fruit fly species. It can survive in cooler climates [11]. The vetch aphid (*Megoura viciae* L.) is an insect whose life cycle is mainly associated with the decomposition of legumes, especially *Lathyrus* L., vetch, pea, and broad bean [12]. *Caenorhabditis elegans* L. is a free-living, transparent nematode that lives in soil and rotting fruit [13]. Another insect is the tobacco budworm (*Heliothis virescens* L.), which attacks crops such as soybeans, cotton, flax, tobacco, vegetables, and flowers [14]. The green peach aphid (*Myzus persicae* L.) is a field pest that attacks ornamentals and vegetables grown in greenhouses. It can also transmit plant viruses [15]. The greenhouse whitefly (*Trialeurodes vaporariorum* L.) is a pest of crops such as cucumber, tomato, pepper, eggplant, and ornamentals [16]. The yellow fever mosquito (*Aedes aegypti* L.) is a widespread pest, particularly in subtropical and tropical regions, that transmits viruses causing diseases such as Zika, dengue, and yellow fever [17]. Some fungi, such as *Phytophthora infestans*, *Zymoseptoria tritici*, *Botrytis cinerea*, and *Fusarium culmorum*, can cause significant damage to agriculture and human health. *Phytophthora infestans* is responsible for late blight or potato blight. *Zymoseptoria tritici*, a wheat pathogen, causes *Septoria* leaf blotch, one of the most important wheat diseases today. This disease is difficult to control due to resistance to several fungicides [10]. *Botrytis cinerea*, commonly known as ‘gray mold’, is a necrotrophic fungus that can affect a variety of plant species, including grapes. *Fusarium culmorum*, a fungal plant pathogen, can cause damage such as seedling blight, foot rot, ear rot, stem rot, common root rot, and other diseases in grasses, monocots, and dicots.

If these species become resistant to pesticides, finding alternative solutions to protect agriculture and crops is crucial. At the same time, reducing pesticide use is a crucial goal in achieving sustainable agriculture. Studies on pesticide reduction have typically compared a limited number of experimental prototypes [2]. One solution is to explore natural, biologically active products and their derivatives. Natural compounds are less harmful to both humans and the environment than many synthetic compounds. Therefore, they may be good candidates for more effective and less toxic herbicides, insecticides and fungicides than toxic synthetic alternatives.

One natural compound used for many years as a herbicide that was approved for sale by the EPA in 1992, and approved by the FDA as safe for human consumption in small amounts, is pelargonic acid, also known as nonanoic acid [18]. The sodium and potassium salts of unsaturated fatty acids, which are harmless to humans, can be used as insecticides against *Aphididae* species [19]. Additionally, certain bacteria and parasitic nematodes can serve as bioinsecticides [20]. These natural compounds provide a safe alternative to synthetic pesticides, reassuring us about their use in agriculture.

Naturally occurring small molecules are organic compounds with diverse biological activities. Alkaloids are a type of such compounds. This large group of essential compounds includes indole derivatives like gramine, troponoids like colchicine, and purine compounds like caffeine. These alkaloids and their derivatives are biologically active organic compounds of great importance, particularly in pharmacology. In addition to medicine, these compounds have potential applications in industry and agriculture to minimize weed infestation and act as insecticides and fungicides. Colchicine and caffeine have been considered as potential herbicides due to their biological effects [21]. Gramine and its analogs are known to exhibit antiviral activity in plants. Gramine repels many herbivorous insects, including the grasshopper (*Locusta migratoria* L.), the wheat weevil (*Schizaphis graminum* L.), the corn aphid (*Rhopalosiphum padi* L.), and the aphid (*Sitobion avenae* L.) [22]. This alkaloid also disrupts the oxidation-reduction processes in cyanobacterial cells, leading to their death. Compared to other allelochemicals, gramine is one of the most potent allelochemicals affecting the development of *M. aeruginosa* L. [23,24]. The antifouling effect of naturally occurring 2,5,6-tribromomethylogramine (TBG) is robust and comparable to copper oxide and other known agents [25,26]. Additionally, TBT (5,6-dichloro-1-methylgramine) inhibits the growth of arthropods and mussels [25]. Gramine derivatives also inhibit the reproduction of tobacco mosaic virus [27], and many gramine derivatives have antifungal properties. They are highly active against *T. rubrum* L. and *T. mentagrophytes* L. Caffeine in plants acts as an allelopathic agent and can be used to protect important crops. This alkaloid is both a deterrent and toxic to slugs and snails [28]. It can be used as a natural larvicide against *Ae. aegypti* L. [29]. Some caffeine derivatives act as insecticides against *Mythimna separata Walker* L. and *Culex pipiens pallens* L. and have antifungal activity against some plant pathogenic fungi [30]. Colchicine and its derivatives are known for their antifungal properties [31,32,33,34,35,36,37,38,39].

This broad spectrum of alkaloid activity suggests their potential use in crop protection against phytopathogens, particularly in economically important crops. Testing of alkaloids as potential herbicidal, insecticidal, and fungicidal agents could lead to significant advances in crop protection.

Colchicine, gramine, caffeine, and their derivatives have not been tested as potential agricultural and crop protection agents on the abovementioned weeds, insects, and fungi. This led us to propose a collaborative investigation of these compounds with BASF’s Open Innovation Platform Agro, leveraging its expertise and resources.

## 2. Results and Discussion

Alkaloids, which are small bioactive molecules, may be useful in humans as drugs and as fungicidal, herbicidal, and insecticidal agents [40,41,42]. They are a large group of secondary metabolites explicitly produced by plants [43]. Gramine has many biological activities, including insecticidal, antibacterial, antiviral, fungicidal, algicidal, anti-tumor, and anti-inflammatory activities [44]. Colchicine has anti-inflammatory and anticancer activities, but to date, colchicine and its derivatives have not been tested for their fungicidal, herbicidal, and insecticidal specific pathogens described in this paper. Caffeine, in addition to its known activity on human immune and nervous systems, also has antifungal, antibiofilm, and antioxidant effects [45,46].

The aim of this study was to evaluate selected compounds such as colchicine, gramine, and caffeine and their derivatives as potential herbicidal, fungicidal, and insecticidal agents in the context of their usefulness as agricultural substances and for the protection of crops against specific pathogens.

Alkaloids: colchicine (**1**), colchiceine (**2**), and its derivatives and complexes (**3**–**12**) [31,32,33,34,35,36,37,38], gramine (**13**) and its derivatives (**14**–**34**), and caffeine (**35**) and its derivatives (**36**–**39**, **48**) were tested as potential herbicidal, insecticidal, and fungicidal agents. The structures of all compounds are presented in Table 1, Table 2 and Table 3. All compounds were confirmed to be >95% pure, and their structures were confirmed by spectroscopic methods (NMR, FT IR).

BASF investigated the efficacy of the tested compounds against fungal stains, including *Phytophthora infestans*, *Zymoseptoria tritici*, *Botrytis cinerea*, and *Fusarium culmorum*, as well as insects such as Boll weevil (*Anthonomus grandis*), Mediterranean fruit fly (*Ceratitis capitata*), Vetch aphid (*Megoura viciae*), Caenorhabditis elegans, and tobacco budworm (*Heliothis virescens*). The study included the green peach aphid (*Myzus persicae*), greenhouse whitefly (*Trialeurodes vaporariorum*), yellow fever mosquito (*Aedes aegypti*), and weeds such as *Capsella*, *Agrostis stolonifera*, and *Poa annua*.

Given the significant threat these insect, fungal, and weed species pose to crop and agricultural productivity, it is crucial to test new compounds for crop protection. We have identified natural alkaloids such as colchicine, gramine, caffeine, and their derivatives as potential biologically active compounds due to their small molecule size and promising crop protection potential.

BASF procedures consisted of preliminary screening of all compounds in silico using the company’s system to identify possible biologically active molecules. The scores used to indicate the activity of the tested compounds are described as follows: 1 for active, 2 for average activity, and 3 for not active.

After preliminary screening by in silico methods, 14 compounds that are gramine derivatives (compounds **13**–**15**, **17**, **26**–**35**) were selected from 94 compounds and used for further biological testing as fungicides, insecticides, and herbicides. Only five compounds showed fungicidal activity against *Botrytis cinerea*, *Fusarium culmorum*, *Phytophthora infestans*, and *Zymoseptoria tritici*. All 14 compounds tested biologically showed no herbicidal or insecticidal activity.

Table 4 presents the antifungal activity of five gramine derivatives: **14**, **27**, **29**, **31**, and **35**. Compound **35** exhibited the highest antifungal activity against four pathogenic fungi. The other derivatives showed moderate activity against *Botrytis cinerea* (**27**,**31**), *Phytophthora infestans* (**14**), *Zymoseptoria tritici* (**14**,**31**), and *Fusarium culmorum* (**29**). Compound **29** also showed moderate activity against *Botrytis cinerea* and *Zymoseptoria tritici*. Regarding the activity of gramine derivatives, the length and branching of the introduced alkyl chain are crucial factors. A comparison of the activity of compounds **27**–**32** shows differences in their ether groups. The derivatives with ethoxy (**27**) and butoxy (**29**) groups show activity, while those with propoxy (**28**) and pentoxy (**30**) groups do not. Compound **31**, a gramine derivative with a branched alkyl chain, is also active. The pyrrolidine dithiocarbamate moiety in the most active compound, **35**, seems crucial for the biological activity of the tested derivatives.

To understand why certain compounds exhibited higher activity than others, we performed in silico tests to predict key parameters such as lipophilicity, hydrophobicity (water solubility), and toxicity. These parameters are critical for understanding the interaction of the compounds with pathogens, while also considering their potential use in agriculture and crop protection.

The SwissADME web tool was used to predict the water solubility of the tested compounds.

Water solubility is a critical physical property in medicinal and agrochemical chemistry because it affects the distribution and uptake of biologically active compounds in living cells, tissues, organisms, and the environment. It is essential to note that optimal distribution and uptake of these compounds require good water solubility. When considering chemicals that may be washed into waterways, it is crucial to balance agricultural benefits with potential environmental impact.

Compounds can be classified based on their solubility values (LogS). Highly soluble compounds have solubility values of 0 or higher, while those in the range of 0 to −2 are considered soluble. Compounds with solubility values of −2 to −4 are slightly soluble, and those with solubility values less than −4 are insoluble.

Table 5 displays LogS values obtained from ESOL, Ali, and SILICOS-IT methods. According to the data given in Table 5, all 14 compounds are either soluble or moderately soluble. Compound **35**, the most active one, is less soluble than the starting compound **13** (gramine). Compounds **14**, **27**, **29**, and **31**, which are also active, are more soluble than compound **35**.

Lipophilicity is a crucial factor affecting molecular interactions, leading to ligand-receptor binding or enzyme inhibition potency modifications. It is also a property that facilitates passive drug transport. The lipophilicity of all 14 compounds was predicted using three different web tools (MolInspiration, SwissADME, and Protox II) to enable data comparison.

The results are summarized in Table 6. Table 6 shows the LogP values of the tested compounds, indicating an increase in lipophilicity for the gramine derivatives compared to the parent gramine (**13**). Specifically, compounds **14**, **27**, and **31** have LogP values (obtained from MolInspiration) in the range of 2.42–2.78, while compounds **29** and **35** have logP values of 3.42 to 3.48, indicating significantly higher lipophilicity than **13**.

The SwissADME web tool offers five methods (iLOGP, XLOGP3, WLOGP, MLOGP, SILICOS-IT) to predict lipophilicity of compounds, as well as a Consensus LogP parameter, which is the average value of all five predictions (LogP^2^–LogP^7^). The LogP values calculated for the tested compounds (Table 6) indicate an increase in lipophilicity of the gramine derivatives compared to the gramine (**13**).

The LogP values calculated by the Protox II platform (LogS^8^) were slightly higher but followed the same trend.

As part of the MolInspiration tool, we used Galaxy Visualizer 3D to display the molecular lipophilicity potential (MLP) on the molecular surface (see Table 7).

We compared the tested derivatives by visualizing their molecules as structural formulas and obtained the molecular surface for space-filling CPK models using Galaxy Generator 3D.

Nowadays, it is essential for chemical compounds with potential practical applications to be environmentally friendly and safe for human use. When evaluating these compounds, it is essential to consider their toxicity and LD_50_ values, particularly in the case of oral administration. To assess the toxicity of the 14 compounds tested, we used the Protox II web tool and present our predictions’ oral toxicity results in Table 8. Acute toxicity of compounds is measured on a scale from 1 to 6, with 1 indicating substantial toxicity and 6 indicating non-toxicity. Of the 14 compounds tested, 12 were predicted to have a toxicity level of 4 (harmful if swallowed). Compound **28** was predicted to have a toxicity level of 5 (may be harmful if swallowed) but was inactive in biological tests. Compound **35** was found to be non-toxic. The results indicate that the starting alkaloid, gramine (**13**), is significantly more toxic than the derivatives obtained, with a predicted LD_50_ value of 380 mg/kg. Among the compounds that exhibited antifungal activity (**14**, **27**, **29**, **31**, and **35**), compound **14** had the highest predicted LD_50_ value (500 mg/kg), followed by compound **29** (LD_50_ = 1000 mg/kg), and compounds **27** and **31** (LD_50_ = 1425 mg/kg). Compound **35** demonstrated the highest efficacy against the tested fungal species and exhibited low toxicity with an LD_50_ of 5600 mg/kg. This finding is significant as it suggests that the compound could be utilized in both human and agrochemical environments.

In addition to predicting the LD_50_ values of biologically active derivatives, detailed toxicity predictions were conducted for all 14 compounds. The predictions suggest that all 14 compounds are hepatotoxic and inactive. Regarding other toxicity endpoints, such as carcinogenicity, immunotoxicity, mutagenicity, and cytotoxicity, compounds **17** and **28** were carcinogenic, and compounds **17** and **34** were mutagenic.

The Protox II platform utilizes the Tox21 protocols to develop novel methods for assessing chemical toxicity. This platform could enhance the evaluation of environmental chemicals and the development of new medicines. Chemical compounds can disrupt processes in the human body, which can result in adverse health effects. According to this model, two aspects are essential: nuclear receptor signaling and stress response pathways.

The nuclear receptor signaling pathways predictions for all 14 gramine derivatives involve seven different receptors: aryl hydrocarbon receptor (AhR), androgen receptor (AR), androgen receptor ligand binding domain (AR-LBD), aromatase, estrogen receptor alpha (ER), estrogen receptor ligand binding domain (ER-LBD), and peroxisome proliferator-activated receptor gamma (PPAR-Gamma). Only compounds **15**, **27**, and **31** were found to be active in the aryl hydrocarbon receptor (AhR). All other compounds were inactive in all seven receptors. All 14 compounds were inactive in the predictions of Tox21 stress response pathways with five receptors: nuclear factor (erythroid-derived 2)-like 2/antioxidant responsive element (nrf2/ARE), heat shock factor response element (HSE), mitochondrial membrane potential (MMP), phosphoprotein (tumor suppressor) p53, and ATPase family AAA domain-containing protein 5 (ATAD5).

## 3. Materials and Methods

### 3.1. Compound Preparation

Colchicine is commercially available from Sigma Aldrich. Gramine is commercially available from Sigma Aldrich. Caffeine is commercially available from Sigma Aldrich. Colchicine complexes were prepared according to [31,32,33,34,39]. Colchiceine and complexes were prepared according to [32,35]. 10-Alkylthiocolchicines were prepared according to [36]. 7-Deacetyl-10-alkylthiocolchicines were prepared according to [37]. Complexes of 10-methylthiocolchicine were prepared according to [38]. Gramine derivatives were prepared according to literature procedures: **1**–**14** [47], **15**–**23** [49], **25** and **27**–**32** [50], **26** [48], **33** [51], **34** [52], **35** [49]. Caffeine derivatives were prepared according to **36**–**44** [50], **44**–**45** [48] and 47–48 [51].

### 3.2. In Silico Screening/Calculations

A KNIME workflow was used to automate the virtual screening of the compounds using a predefined, indication-specific, knowledge-based set of property value cut-offs.

These included logP (the *n*-octanol-water partition coefficient), logS (unit stripped logarithm (base 10) of the solubility measured in mol/liter), molecular weight, acceptors and donors, and proprietary filters of BASF. Based on these filters, the compounds were either rejected or assigned to the corresponding indication for testing in our preliminary screens.

Web tools were used to predict parameters such as water solubility, lipophilicity, toxicity, LD_50_ values, and other factors and physicochemical properties for all 14 tested compounds. The MolInspiration Cheminformatics tool was used with ALOGPS 2.1. The Virtual Computational Chemistry Laboratory was used for LogP calculations (data given in Table 6), and the MolInspiration Galaxy 3D generator (data given in Table 7) and Galaxy Visualizer were used to visualize the molecular lipophilicity potential (MLP) on the molecular surface (Table 8).

The SwissADME web tool was also used to predict the lipophilicity LogP values (by five models) of the 14 compounds chosen for biological tests and to predict water solubility with LogS values. The data are summarized in Table 5 and Table 6.

The ProTox-II platform is divided into five different classification steps: 1. acute toxicity (oral toxicity model with six different toxicity classes 1–6); 2. organ toxicity (one model, hepatotoxicity); 3. toxicological endpoints (four models); 4. toxicological pathways (12 models), and 5. toxicity targets (15 models). The platform makes predictions on LD_50_ values. It used Tox21 (the Toxicology in the 21st Century program, a federal collaboration involving NIH, the Environmental Protection Agency, and the Food and Drug Administration, aims to develop better toxicity assessment methods).

### 3.3. Fungicidal Tests

Fungi Strains: *Phytophthora infestans*, *Zymoseptoria tritici*, *Botrytis cinerea*, *Fusarium culmorum*. The growth assay was performed in 96-well plates, 1 concentration (31 ppm), 3 technical replicates. Layout: Column 1—DMSO (negative control) + spore suspension, column 12—positive control + spore suspension, columns 2–10—compounds + spore suspension. Test duration 4–7 days. Conditions: 22.9 °C, humidity 90–100%. Raw data: Optical density measured at 620 nm. Algorithmic conversion to activity (growth inhibition). Microtest/growth assay. The active compounds were formulated separately as a stock solution with a concentration of 10,000 ppm in dimethyl sulfoxide.

#### 3.3.1. Activity against Phytophthora Infestans in the Microtiter Plate Assay

The stock solutions were mixed according to the ratio, pipetted onto a microtiter plate (MTP) and diluted with water to the indicated concentrations. A spore suspension of *Phytophthora infestans* in pea solution was then added. The plates were placed in a chamber at 22.9 °C and 90–100% humidity. Using an absorbance photometer, MTPs were measured at 620 nm every 15 h for 6 days after inoculation. The measured parameters were compared with the growth of the control without active ingredient (100%) and the fungus-free and active-ingredient-free blank to determine the relative growth percentage of the pathogens in the respective active ingredients.

#### 3.3.2. Microtiter Plate Activity against Zymoseptoria Tritici

The stock solutions were mixed according to the ratio, pipetted onto a microtiter plate (MTP), and diluted with water to the indicated concentrations. A spore suspension of *Zymoseptoria tritici* in malt solution was then added. The plates were placed in a chamber at 22.9 °C and 90–100% humidity. Using an absorbance photometer, MTPs were measured at 620 nm every 8 h starting 2 days after inoculation (test duration was 4 days). The measured parameters were compared with the growth of the control without active ingredient (100%) and the fungus-free and active-ingredient-free blank to determine the relative growth percentage of the pathogens in each active ingredient.

#### 3.3.3. Microtiter Plate Activity against Botrytis Cinerea

The stock solutions were mixed according to the ratio, pipetted onto a microtiter plate (MTP), and diluted with water to the indicated concentrations. A spore suspension of *Botrytis cinerea* in malt solution was then added. The plates were placed in a chamber at 22.9 °C and 90–100% humidity. Using an absorbance photometer, MTPs were measured at 620 nm every 15 h for 6 days after inoculation. The measured parameters were compared with the growth of the control without active ingredient (100%) and the fungus-free and active-ingredient-free blank to determine the relative growth percentage of the pathogens in the respective active ingredients.

#### 3.3.4. Activity against Fusarium Culmorum in the Microtiter Plate Test

The stock solutions were mixed according to the ratio, pipetted onto a microtiter plate (MTP), and diluted with water to the indicated concentrations. A spore suspension of *Fusarium culmorum* in YBG solution was then added. The plates were placed in a chamber at 22.9 °C and 90–100% humidity. Using an absorbance photometer, MTPs were measured at 620 nm every 15 h for 5 days after inoculation. The measured parameters were compared with the growth of the control without active ingredient (100%) and the fungus-free and active-ingredient-free blank to determine the relative growth percentage of the pathogens in the respective active ingredients.

### 3.4. Insecticidal Tests

#### 3.4.1. Test to Evaluate the Control of Vetch Aphid (*Megoura viciae*) by Contact or Systemic Means

The test unit consisted of 24-well microtiter plates containing broad bean leaf discs. The compounds were formulated using a solution containing 75% water and 25% DMSO. Different concentrations of the formulated compounds or mixtures were sprayed onto the leaf discs at 2.5 µL using a custom-built microsprayer in two replicates. After application, the leaf discs were air dried and 5–8 adult aphids were placed on the leaf discs in the microtiter plate wells. The aphids were then allowed to suck on the treated leaf discs and incubated at 23 + 1 °C, 50 + 5% RH for 5 days. Aphid mortality and fecundity were then assessed visually.

#### 3.4.2. Test to Evaluate the Control of *Caenorhabditis elegans* by Contact or Systemic Means

The test unit consisted of 96-well microtiter plates containing a liquid diet. The compounds were formulated using a solution containing 75% water and 25% DMSO. Different concentrations of formulated compounds or mixtures were sprayed into the microtiter plate wells at 5 µL per well using a custom-built microsprayer in two replicates. Mixed instar 60–100 *C. elegans* were transferred to the microtiter plate wells. After application, nematodes were incubated at 18 + 1 °C, 70 + 5% RH for 4 days. Nematode motility (mortality) was then assessed visually.

#### 3.4.3. Test to Evaluate the Control of Green Peach Aphid (*Myzus persicae*) by Systemic Means

The test unit consisted of 96-well microtiter plates containing liquid artificial diet under an artificial membrane. The compounds were formulated using a solution containing 75% water and 25% DMSO. Different concentrations of formulated compounds or mixtures were pipetted into the aphid diet using a custom-designed pipette in two replicates. After application, 5 to 8 adult aphids were placed on the artificial membrane within the wells of the microtiter plate. The aphids were then allowed to feed on the treated aphid diet and incubated at 23 + 1 °C, 50 + 5% RH for 3 days. Aphid mortality and fecundity were then assessed visually.

#### 3.4.4. Boll Weevil (*Anthonomus grandis*) Control Test

The test unit consisted of 96-well microtiter plates containing insect food and 20–30 *A. grandis* eggs. The compounds were formulated using a solution containing 75% water and 25% DMSO. Different concentrations of the formulated compounds or mixtures were sprayed onto the insect diet at 5 µL using a custom-built microsprayer in two replicates. After application, microtiter plates were incubated at 23 + 1 °C, 50 + 5% RH for 5 days. Egg and larval mortality were then assessed visually.

#### 3.4.5. Test to Evaluate the Control of Mediterranean Fruit Fly (*Ceratitis capitata*)

The test unit consisted of 96-well microtiter plates containing insect food and 50–80 *C. Capitata* eggs. The compounds were formulated using a solution containing 75% water and 25% DMSO. Different concentrations of the formulated compounds or mixtures were sprayed onto the insect diet at 5 µL using a custom-built microsprayer in two replicates. After application, microtiter plates were incubated at 28 + 1 °C, 80 + 5% RH for 5 days. Egg and larval mortality was then assessed visually.

#### 3.4.6. Test to Evaluate the Control of Tobacco Budworm (*Heliothis virescens*)

The test unit consisted of 96-well microtiter plates containing insect food and 15–25 *H. virescens* eggs. The compounds were formulated using a solution containing 75% water and 25% DMSO. Different concentrations of the formulated compounds or mixtures were sprayed onto the insect diet at 10 µL using a custom-built micro nebulizer in two replicates. After application, microtiter plates were incubated at 28 + 1 °C, 80 + 5% RH for 5 days. Egg and larval mortality was then assessed visually.

#### 3.4.7. Test to Evaluate the Control of Tobacco Budworm Yellow Fever Mosquito (*Aedes aegypti*) Control Assay

The assay device consisted of 96-well microtiter plates containing 200 µL of tap water per well and 5–15 freshly hatched *A. aegypti* larvae. The compounds were formulated using a solution containing 75% water and 25% DMSO. Different concentrations of formulated compounds or mixtures were sprayed onto the insect food at 2.5 µL using a custom-built microsprayer in two replicates. After application, microtiter plates were incubated at 28 + 1 °C, 80 + 5% RH for 2 days. Larval mortality was then assessed visually.

#### 3.4.8. Test to Evaluate Control of Greenhouse Whitefly (*Trialeurodes vaporariorum*)

The test unit consisted of 96-well microtiter plates containing a leaf disk of eggplant with whitefly eggs. The compounds were formulated using a solution containing 75% water and 25% DMSO. Different concentrations of formulated compounds or mixtures were sprayed onto the leaf discs at 2.5 µL using a custom-built microsprayer in two replicates. After application, microtiter plates were incubated at 23 + 1 °C, 65 + 5% RH for 6 days. Mortality of hatched caterpillars was then assessed visually.

### 3.5. Herbicidal Test

The compounds were dissolved in a 1:10 mixture of DMSO and water containing 1% of the wetting agent Dash. These solutions were then homogenized and sprayed onto plants and seeds in trays using an ultrasonic spray head at an application rate equivalent to 2 kg/ha. The three plant species used in the test were *Agrostis stolonifera*, *Poa annua*, and *Matricaria inodora*. For each species, an application was made to the growing plants (post-emergence) and to the ungerminated seeds in the soil (pre-emergence). Herbicidal activity was evaluated after seven days on a scale from 0% (no activity) to 100% (complete kill), using plants growing in the same trays treated with blank and standard herbicide solutions.

## 4. Conclusions

Our preliminary studies on the fungicidal, herbicidal, and insecticidal activity of alkaloids suggest that they could be a promising group of natural compounds for the development of effective, non-toxic crop protection agents. The most promising results were obtained with gramine derivatives (indole derivatives). Five of the gramine derivatives were active against microfungal species, and their activity was influenced by the type of group attached through the methylene bridge to the C3 position of the indole system. The gramine derivative containing a pyrrolidine dithiocarbamate moiety showed the most promising results. It showed significant activity against four fungal species due to its unique composition and resulting physicochemical properties in biological interactions with chemical compounds, which distinguish it from the other compounds. Furthermore, the most active in the fungicidal tests, the gramine derivatives, showed the lowest water solubility, lipophilicity, and toxicity among the tested compounds, indicating their potential for future use as crop protection agents.

## Figures and Tables

**Table 1:** Colchicine derivatives **1–12**.

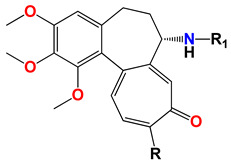	**No.**	**R**	**R_1_**	**Ref.**
	**1**	-OCH_3_ colchicine	-COCH_3_	-
	**2**	-OH colchiceine	-COCH_3_	[35]
	**3**	-SCH_3_	-COCH_3_	[36]
	**4**	-SCH_2_CH_3_	-COCH_3_	[36]
	**5**	-SCH_2_CH_2_CH_3_	-COCH_3_	[36]
	**6**	-SCH(CH_3_)_2_	-COCH_3_	[36]
	**7**	-SCH_2_CH_2_CH_2_CH_3_	-COCH_3_	[36]
	**8**	-SCH_3_	-H	[37]
	**9**	-SCH_2_CH_3_	-H	[37]
	**10**	-SCH_2_CH_2_CH_3_	-H	[37]
	**11**	-SCH(CH_3_)_2_	-H	[37]
	**12**	-SCH_2_CH_2_CH_2_CH_3_	-H	[37]

**Table 2:** Gramine and tested derivatives **13**–**34**.

Compounds	No.	R_1_	R_2_	Ref.
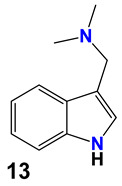 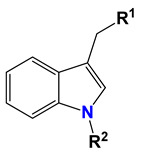	**13**	-N(CH_3_)_2_ gramine	-H	
	**14**	-OCOCH_3_	-COCH_3_	[47]
	**15**	-OC CH	-H	[47]
	**16**	-OCH_2_CH_3_	-(CH_2_)_3_Br	[47]
	**17**	-OCH_2_CH_3_	-(CH_2_)_3_N_3_	[47]
	**18**	-OCH_2_CH_3_	-(CH_2_)_5_Br	[47]
	**19**	-OCH_2_CH_3_	-(CH_2_)_5_N_3_	[47]
	**20**	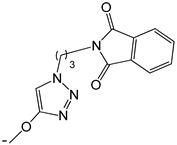	-H	[47]
	**21**	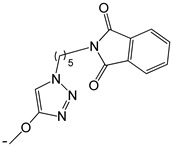	-H	[47]
	**22**	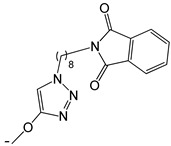	-H	[47]
	**23**	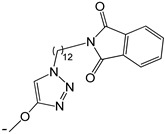	-H	[47]
	**24**	-OC_2_H_5_	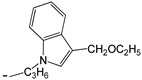	[47]
	**25**	-OC_2_H_5_	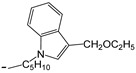	[47]
	**26**	-OH	-COCH_3_	[48]
	**27**	-OC_2_H_5_	-H	[49]
	**28**	-OCH_2_CH_2_CH_3_	-H	[49]
	**29**	-OCH_2_CH_2_CH_2_CH_3_	-H	[49]
	**30**	-OCH_2_CH_2_CH_2_CH_2_CH_3_	-H	[49]
	**31**	-OCH(CH_3_)_2_	-H	[49]
	**32**	-OCH_2_CH_2_CH(CH_3_)_2_	-H	[49]
	**33**	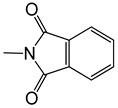	-H	[49]
	**34**	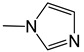	-H	[49]
	**35**	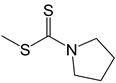	-H	[49]

**Table 3:** Caffeine and derivatives **35–48**.

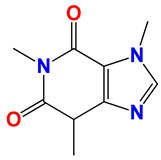 Caffeine 36 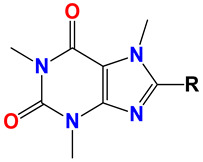	**No.**	**R_1_**	**Ref.**
	**36**	-H	-
	**37**	-Br	[50]
	**38**	-N_3_	[51]
	**39**	-SCH_3_	[50]
	**40**	-SC_2_H_5_	[50]
	**41**	-SCH_2_CH_2_CH_3_	[50]
	**42**	-SCH(CH_3_)_2_	[50]
	**43**	-SC(CH_3_)_3_	[50]
	**44**	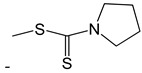	[50]
	**45**	-NHNH_2_	[52]
	**46**	-NH(CH_2_)_2_NH_2_	[48]
	**47**	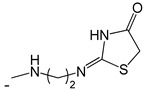	[53]
	**48**	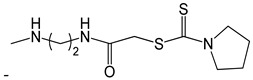	[53]

**Table 4:** Activity of selected 14 compounds against for selected four fungi stains, activity score: 1—active, 2—average activity, 3—not active.

Comp. No.	*Fungi Stains*			
	*Botrytis* *cinerea*	*Fusarium* *culmorum*	*Phytophthora infestans*	*Zymoseptoria tritici*
**13**	3	3	3	3
**14**	3	3	2	2
**15**	3	3	3	3
**17**	3	3	3	3
**26**	3	3	3	3
**27**	2	3	3	3
**28**	3	3	3	3
**29**	1	2	3	1
**30**	3	3	3	3
**31**	2	3	3	2
**32**	3	3	3	3
**33**	3	3	3	3
**34**	3	3	3	3
**35**	1	1	2	1

**Table 5:** Water solubility of 14 tested compounds predicted by the SwissADME web tool.

Comp.	Water Solubility		
	LogS (ESOL) [54] /Class	Log S (Ali) [55] /Class	Log S (SILICOS-IT) [56] /Class
**13**	−2.42/soluble	−1.80/very soluble	−3.79/soluble
**14**	−2.39/soluble	−2.19/soluble	−3.28/soluble
**15**	−2.69/soluble	−2.39/soluble	−3.51/soluble
**17**	−3.35/soluble	−4.22/moderate soluble	−4.76/moderate soluble
**26**	−1.99/very soluble	−1.48/very soluble	−2.63/soluble
**27**	−2.48/soluble	−2.11/soluble	−4.22/moderate soluble
**28**	−2.79/soluble	−2.66/soluble	−4.63/moderate soluble
**29**	−3.01/soluble	−3.03/soluble	−5.04 moderate soluble
**30**	−3.34/soluble	−3.59/soluble	−5.44/moderate soluble
**31**	−2.80/soluble	−2.57/soluble	−4.25 moderate soluble
**32**	−3.35/soluble	−3.49/soluble	−5.07/moderate soluble
**33**	−3.54/soluble	−3.28/soluble	−5.87/moderate soluble
**34**	−2.65/soluble	−1.95/very soluble	−4.22/moderate soluble
**35**	−3.73/soluble	−4.56/moderate soluble	−4.39/moderate soluble

**Table 6:** Lipophilicity of 14 tested compounds predicted by MolInspiration, SwissADME, and Protox II web tools.

Comp.	Lipophilicity							
	LogP [57]	LogP (iLOGP) [58]	LogP (XLOGP3) [59]	LogP (WLOGP) [60,61,62]	LogP (MLOGP) [63]	LogP (SILICOS-IT) [64]	Consensus LogP [65]	Log P [66]
**13**	1.89	1.88	1.78	2.08	1.55	2.41	1.94	2.23
**14**	2.45	2.43	1.57	2.21	1.84	2.06	2.02	2.36
**15**	1.82	2.03	2.23	2.20	1.47	3.00	2.19	2.28
**17**	3.46	3.02	3.21	3.73	1.27	1.98	2.64	3.33
**26**	1.75	1.77	1.00	1.64	1.40	1.64	1.49	1.79
**27**	2.42	2.01	1.96	2.55	1.55	3.18	2.25	2.7
**28**	2.92	2.26	2.49	2.94	1.84	3.53	2.61	3.09
**29**	3.48	2.58	2.85	3.33	2.11	3.89	2.95	3.48
**30**	3.98	2.85	3.39	3.72	2.37	4.26	3.32	3.87
**31**	2.78	2.29	2.40	2.94	1.84	3.35	2.56	3.09
**32**	3.69	2.67	3.29	3.58	2.37	4.09	3.20	3.73
**33**	3.48	2.05	2.52	2.43	2.75	3.41	2.63	2.90
**34**	1.95	1.48	1.63	2.41	1.16	2.42	1.82	2.41
**35**	3.42	2.61	3.28	3.25	2.29	4.63	3.21	3.72

**Table 7:** Visual comparison of the hydrophobic (blue) and hydrophilic (orange and red) parts of the tested compounds’ molecules present through space-filling CPK models.

Compounds Structures	Dotted Models	Space-Filling CPK Models
Gramine **13** 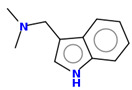	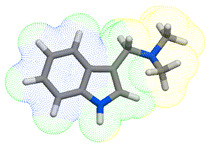 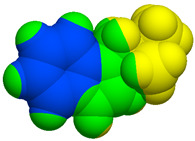	
**14** 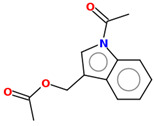	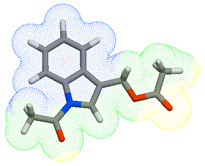 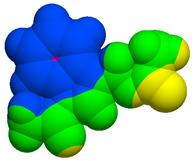	
**15** 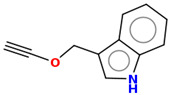	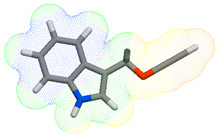 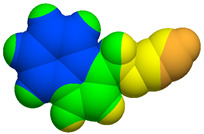	
**17** 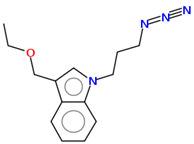	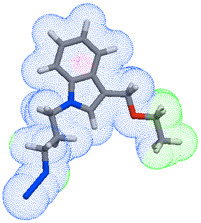 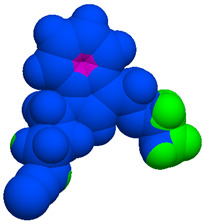	
**26** 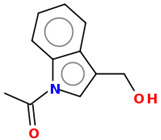	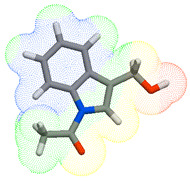 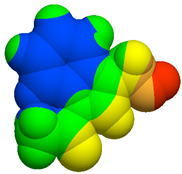	
**27** 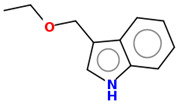	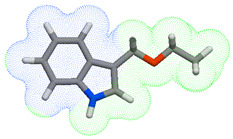 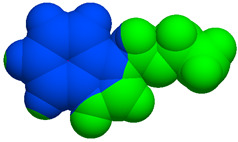	
**28** 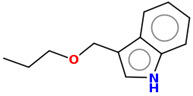	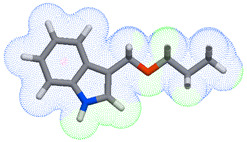 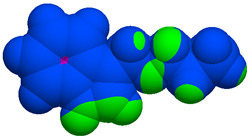	
**29** 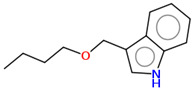	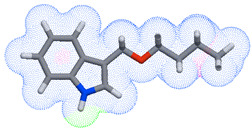 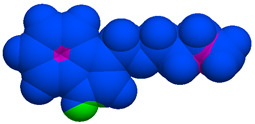	
**30** 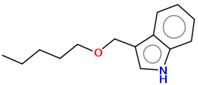	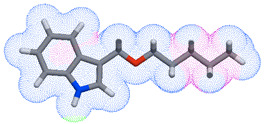 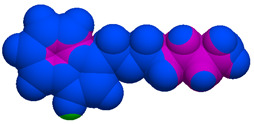	
**31** 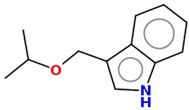	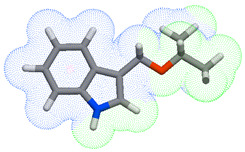 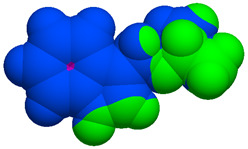	
**32** 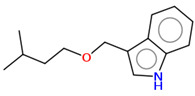	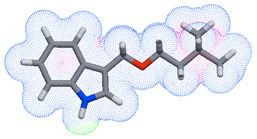 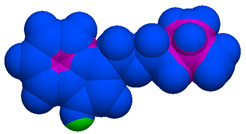	
**33** 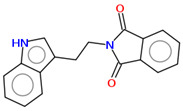	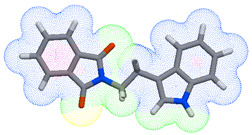 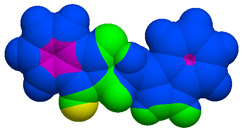	
**34** 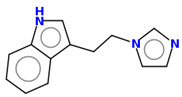	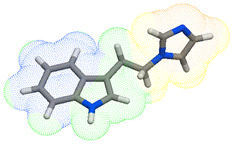 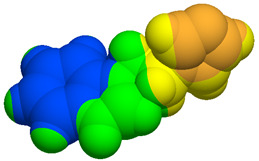	
**35** 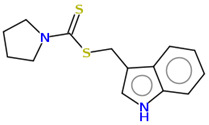	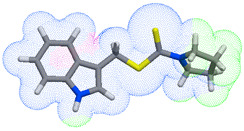 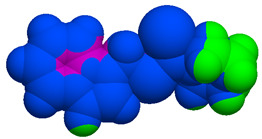	

**Table 8:** Oral toxicity prediction results obtained by Protox II tool [64].

Comp.	Molecular Weight	Number of Hydrogen Bond Acceptors	Number of Hydrogen Bond Donors	Number of Atoms	Number of Bonds	Number of Rotable Bonds	Molecular Refractivity	Topological Polar Surface Area	Predicted LD_50_ mg/kg	Precicted Toxicity Active 1–6 Inactive
**13**	174.24	15	1	27	28	2	56.77	19.03	380	4
**14**	231.25	16	0	30	31	4	64.29	48.3	500	4
**15**	171.2	10	1	22	23	2	52.13	25.02	1420	4
**17**	258.32	20	0	37	38	7	73.3	63.91	500	4
**26**	189.21	13	1	25	26	2	54.56	42.23	500	4
**27**	175.23	14	1	26	27	3	53.93	25.02	1425	4
**28**	189.25	16	1	29	30	4	58.77	25.0	5000	5
**29**	203.28	18	1	32	33	5	63.58	25.02	1000	4
**30**	217.31	20	1	35	36	6	68.38	25.02	1000	4
**31**	189.25	16	1	29	30	3	58.77	25.02	1425	4
**32**	217.31	20	1	35	36	5	68.38	25.02	1000	4
**33**	276.29	15	1	33	36	2	83.06	53.17	2000	4
**34**	197.24	12	1	26	28	2	59.83	33.61	1000	4
**35**	276.42	19	1	34	36	4	87.38	76.42	5600	6

## Data Availability

The data that support the findings of this study are available from the corresponding author upon reasonable request.
